# An evolutionary perspective on marine invasions

**DOI:** 10.1111/eva.12906

**Published:** 2020-01-03

**Authors:** April M. H. Blakeslee, Tereza Manousaki, Katerina Vasileiadou, Carolyn K. Tepolt

**Affiliations:** ^1^ Department of Biology East Carolina University Greenville NC USA; ^2^ Institute of Marine Biology, Biotechnology and Aquaculture Hellenic Centre for Marine Research Thalassocosmos Greece; ^3^ Department of Ecology Charles University Prague Czech Republic; ^4^ Department of Biology Woods Hole Oceanographic Institution Woods Hole MA USA

**Keywords:** adaptation, estuarine, evolutionary history, host–parasite interactions, introduction, non‐native, reproduction, sea

## Abstract

Species distributions are rapidly changing as human globalization increasingly moves organisms to novel environments. In marine systems, species introductions are the result of a number of anthropogenic mechanisms, notably shipping, aquaculture/mariculture, the pet and bait trades, and the creation of canals. Marine invasions are a global threat to human and non‐human populations alike and are often listed as one of the top conservation concerns worldwide, having ecological, evolutionary, and social ramifications. Evolutionary investigations of marine invasions can provide crucial insight into an introduced species’ potential impacts in its new range, including: physiological adaptation and behavioral changes to exploit new environments; changes in resident populations, community interactions, and ecosystems; and severe reductions in genetic diversity that may limit evolutionary potential in the introduced range. This special issue focuses on current research advances in the evolutionary biology of marine invasions and can be broadly classified into a few major avenues of research: the evolutionary history of invasive populations, post‐invasion reproductive changes, and the role of evolution in parasite introductions. Together, they demonstrate the value of investigating marine invasions from an evolutionary perspective, with benefits to both fundamental and applied evolutionary biology at local and broad scales.

## 1 INTRODUCTION

Species distributions are rapidly changing, facilitated by enhanced human migration and globalization over the past several centuries. Increasingly, species are becoming introduced to and established in novel locations that are often well outside their limits for natural dispersal. In marine systems, the anthropogenic transfer of living organisms has occurred as a result of multiple mechanisms including shipping, aqua‐ or mariculture, pet trade, bait trade, food trade, and canals. Marine invasions represent a global threat to human populations and broader biological communities and are often listed as one of the top conservation concerns worldwide (IPCC, 2019). As species continue to expand along the coasts and within estuaries, they can have devastating environmental and socioeconomic impacts (Lovell, Stone, & Fernandez, 2006; Bennett, 2018; Giakoumi et al., 2019). Some notable examples include the lionfish *Pterois miles*, the puffer fish *Lagocephalus sceleratus*, the ctenophore *Mnemiopsis leidyi*, the European green crab *Carcinus maenas*, the round goby *Neogobius melanostomus*, and the green alga *Caulerpa taxifolia*. Invasive species can spread rapidly, successfully colonizing and expanding their ranges within a matter of years to decades (e.g., Azzurro, Soto, Garofalo, & Maynou, 2012). In the process, they introduce multiple novel interactions to land and seascapes over a potentially vast new range. Some species may have disproportionately strong effects on their invaded communities, such as habitat‐forming species that greatly alter landscapes postinvasion, for example, global invasions of the macroalga *Agarophyton vermiculophyllum* (formerly *Gracilaria vermiculophylla*) and the Pacific oyster *Crassostrea gigas*. Both add novel structure to soft‐sediment habitats (e.g., Krueger‐Hadfield, Stephens, Ryan, & Heiser, 2018; Reise, 1998; Thomsen et al., 2007; Troost, 2010). Actions that prevent species establishments before they occur are necessary to avoid the destructive damage invasive species can cause: Postestablishment measures to control invasive species often fail and are incredibly costly. However, these preventative strategies can be expensive and impractical, particularly for developing nations (Lodge et al., 2006). Moreover, national strategic planning for marine invasions is globally diverse and typically lacks unified policies across and even within nations (Keller, Geist, Jeschke, & Kühn, 2011). Consequently, rates of anthropogenic transport have continued to increase worldwide, as has the spread and expansion of many species that would otherwise be geographically constrained.

Prevention and control efforts often focus on the ecological and economic harm associated with an invasive species, while the possible evolutionary ramifications of a species invasion are typically overlooked. Yet, investigating marine invasions from an evolutionary perspective can provide crucial insight into an introduced species’ potential influence in its invasive range (Geller, Darling, & Carlton, 2010; Grosholz, 2002; Hänfling, 2007). Evolutionary changes can be rapid and varied, including physiological adaptation and behavioral changes to exploit new environments (Lee, Posavi, & Charmantier, 2012; Piola & Johnston, 2006; Tepolt & Somero, 2014). Likewise, resident species may evolve quickly in response to strong selective forces exerted by invaders, leading to changes in resident populations, community interactions, and even the broader environment (Edgell, Lynch, Trussell, & Palmer, a R., 2009; Faillace & Morin, 2016; Hollander & Bourdeau, 2016). Nonselective forces may also be at play, with the potential for severe reductions in genetic diversity associated with species invasions that influence the resulting genotypic composition in the introduced range (Roman & Darling, 2007). Invasive species may interbreed with native congeners, diluting native genomes and potentially overwhelming evolutionarily novel lineages (Gardner, Zbawicka, Westfall, & Wenne, 2016; Strong & Ayres, 2013). To date, determining how invasion dynamics and species interactions impact evolutionary processes in invaded systems has remained a "black box" for many marine communities.

This special issue focuses on current research advances in the evolutionary biology of marine invasions. The papers presented herein enhance our understanding of the mechanisms of invasion, local adaptation processes, and the interactions of introduced species with native populations in marine environments. The papers in this special issue represent solicited contributions from marine invasion scientists who took part in an inaugural conference called *Marine Evolution 2018* and present research at the forefront of this emerging evolutionary field.

## 2 CONTENT OF THE SPECIAL ISSUE

The papers in this special issue were inspired by *Marine Evolution 2018,* an international conference on evolutionary biology in the marine realm. The conference was assembled by the Center for Marine Evolutionary Biology at the University of Gothenburg, Sweden, and was held in Stromstad, Sweden, from May 15 to 17, 2018. The organizing body (Kerstin Johannesson, Anders Blomberg, Pierre de Wit, Samuel Perini, and Eva Marie Rödström) put out a call for abstracts for special sessions focused on any aspect of marine evolution. We proposed a session on the *Evolutionary Biology of Marine Invasions* and then broadly solicited talks for this session that took place during Session 2 on May 15, 2018, over two time blocks. The talks included a keynote at the beginning of the session by Erik Sotka, followed by talks from: Stacy Krueger‐Hadfield, Leon Green, Carolyn Tepolt, Kirsty Smith, Alexis Simon, Pieternella Lutttikhuizen, Ryan Carnegie, and April Blakeslee. Additionally, a poster session included contributions from Andrew David, Ellika Faust, Iva Popov, Aaren Freeman, Rocío Pérez‐Portela, and Jamie Hudson. Many of the researchers above have contributed papers for this special issue. Finally, keynote speakers gave talks at the start of each day, and one of these keynote speakers, Frederique Viard, is also a contributing author to this special issue.

These papers represent diverse perspectives on the emerging field of evolutionary biology in marine invasions. Early research on marine invasions, and invasion biology more broadly, focused on the significant impacts of non‐native organisms on the ecology of an invaded region (Elton, 1958; Richardson, 2011). More recently, researchers have come to understand that evolutionary change may play a key role both in facilitating species invasions and in mediating the response of the native community to new invaders (Lee & Gelembiuk, 2008; Suarez & Tsutsui, 2008). Similarly, just as invasions have become a natural model for understanding how ecological communities function (Blackburn, 2008), they are attracting more research attention as natural experiments in rapid evolution—particularly relevant in this era of rapidly changing climate (Moran & Alexander, 2014). Evolutionary changes at the population level as the result of marine invasions may then influence the larger community of organisms (both native and non‐native) in the invaded region and even potentially have ecosystem‐level effects, especially for those organisms that play a foundational role (e.g., seaweeds). Papers in this special issue can be broadly classified into a few major avenues of research, which we will introduce in turn: the evolutionary history of invasive populations, postinvasion reproductive changes, and the role of evolution in parasite introductions.

### 2.1 Evolutionary history of invasive populations

Three papers in this special issue use genome‐wide single nucleotide polymorphisms (SNPs) to shed light on the invasion (and preinvasion) evolutionary history of invasive populations. In Australia, Popovic, Matias, Bierne, and Riginos (2019) use genomic data to carefully disentangle divergence and gene flow in *Mytilus* mussels. Their data support the disputed taxon *M. planulatus* as a true native Australian species, which has subsequently admixed with two introduced lineages of *M. galloprovincialis*. These introductions, from distinct populations in the Mediterranean and Atlantic Europe, have interbred with the putative native *M. planulatus* to produce a complex and extensive history of admixture in Australia. This study represents an elegant case study of how genome‐wide markers, when used carefully, can disentangle incomplete historical divergence and contemporary gene flow even in complex, poorly delineated systems. Similarly, Hudson, Johannesson, McQuaid, and Rius (2019) use SNPs to explore divergence and admixture in the tunicate *Ciona intestinalis* in the north Atlantic, both in its native northeast Atlantic and invasive northwest Atlantic ranges. The authors found that the species’ current genetic makeup has been shaped by a complex network of both ancestral and historical divergence and admixture. An ancestral split occurred between native English Channel and deep‐water Sweden populations, with secondary contact between these populations resulting in a distinct native shallow‐water Sweden lineage. The introduced northwest Atlantic population, in turn, appears to be the result of admixture between the native English Channel and shallow‐Sweden lineages. Their work highlights the important role that divergence and admixture, both ancestral and contemporary, may play in the success of introduced marine species. The contribution by Simon et al. (2019) advances understanding of the evolutionary mechanisms behind anthropogenic hybridization. The authors studied hybridization between native species and species introduced by shipping. They detected patterns of hybridization in "dock mussels": the blue mussel *Mytilus edulis* and the Mediterranean mussel *M. galloprovincialis*. They found that these "dock mussels" formed spatially limited hybrid zones with native mussel species at port entrances. The observed homogeneous admixture patterns were restricted to large ports, suggesting early‐stage adaptation and habitat choice combined with limited gene flow, rather than migration selection.

### 2.2 Postinvasion reproductive changes

Species introductions may also have a strong impact on the reproductive biology of an invasive species. Reproductive traits may be the target of postinvasion adaptation to novel environments, or a species’ reproductive strategy may influence its spread in invaded landscapes. Three papers in this special issue examined the influence of reproduction on invasion from an evolutionary context. Green, Havenhand, and Kvarnemo (2019) investigated postinvasion local adaptation to salinity gradients on sperm motility and viability in a highly invasive fish species in Europe. They sought to determine why invasive round goby *Neogobius melanostomus* are absent from fully marine waters even though adults appeared tolerant to these higher salinities. The authors tested whether the fish's absence was due to limitations on sperm function at higher salinities, whether local adaptation to higher or lower salinity waters would differentially affect sperm motility, and whether time since introduction played a role. They found that sperm velocity and the percentage of motile sperm declined in salinity levels higher and lower than those currently experienced by Baltic Sea populations, while performance curves differed depending on collection site and local conditions. Their results suggest that round gobies are under strong selection in their invasive region, indicating ongoing local adaptation or epigenetic acclimation to novel environments. The perspectives piece by Krueger‐Hadfield (2019) discussed the role that life cycle complexity and mating systems play in species invasions. The author argues for the inclusion of mating systems in studies of invasion ecology and evolutionary biology, particularly for species with complex life cycles such as macroalgae. This is because eco‐evolutionary dynamics will differ in organisms with multiple free‐living ploidy stages compared with those which have a single, free‐living ploidy stage (e.g., most plants and animals). Many macroalgae are haplodiplontic, wherein meiosis and fertilization are spatiotemporally separated by haploid and diploid stages. Several aspects of a haplodiplontic species’ biology may strongly influence its impact in invaded communities, including the following: haplodiplontic macroalgae can go through selfing, affecting homozygosity and facilitating invasions; asexual reproduction can lead to the dominance of one ploidy stage and the potential loss of the other stage; and niche differentiation may occur between the haploid and diploid stages. The paper describes in detail the importance of each of these aspects to better understanding marine evolutionary ecology in both native and invaded systems. Le Cam et al. (2019) also focused on macroalgae, exploring the genome‐wide diversity of the emblematic global invader *Sargassum muticum*. Using a microsatellite dataset of ~1,500 individuals from 55 locations throughout its species distribution, the authors observed a remarkable lack of diversity in the introduced populations in contrast to the native populations, suggesting severe founder effects. Further analysis using ddRAD demonstrated a 10‐fold decrease in the genetic diversity of introduced populations, reinforcing the conclusions based on microsatellite analyses. Genetic analyses determined that the European populations likely originated from southern northeast Pacific populations and not northern northeast Pacific populations as previously hypothesized. The authors argue that a critical aspect of the invasion success of partial‐selfing organisms like this seaweed is the strategy for either selfing or outcrossing at different stages of invasion. The authors found that most recent populations tended to outcross while more established introduced populations had a higher preference for selfing. These patterns reflect the emergence of new adaptive genotypes early during the invasion that were then followed by selfing at a later stage, which favored the establishment of advantageous genotypes. Overall, Le Cam et al. (2019) pinpoint a rare case of a successful marine invader using nonclonal reproduction, but with limited genomic diversity.

### 2.3 Evolution and parasites

Although parasites are an often hidden component of many marine ecosystems, they can play a pivotal role in the ecological and evolutionary trajectories of marine and estuarine organisms and their communities. The fact that parasites are often ignored in ecological and evolutionary studies suggests that we are missing critical pieces of the puzzle in understanding these systems. Two papers in the special issue focus on further resolving the importance of parasites to our understanding of marine evolution in invaded ecosystems. Blakeslee et al. (2019) investigated founder effect signatures in species invasions, asking whether these signatures may be more pronounced in parasites versus hosts due to inherent differences in life cycles and host availability. The authors found strong founder effect signatures in four species of trematode parasites that obligately infect a snail host *Tritia obsoleta* in native and non‐native populations along the east and west coasts of North America, respectively. In contrast, the snail host demonstrated little genetic bottlenecking in the introduced range, likely as a result of multiple introduction events and high propagule pressure entrained in the introduction vector (oyster translocations). The parasite final hosts also played a strong role in the resulting patterns, with parasite species using birds as final hosts demonstrating a reduced founder effect compared with those using fish as final hosts. Strong east‐to‐west gene flow was also detected originating from the mid‐Atlantic USA, supporting historical evidence of the putative source location. In the contribution by Tepolt et al. (2019), the authors provide a novel perspective on the evolutionary importance of introduced parasites, by comparing the impact of an invasive castrating parasite on mud crab host populations where host and parasite are coevolved: The host is native, but the parasite is invasive (short‐term coevolution); and the parasite is absent (hosts are naive). This study draws on ecological and meta‐analytical data to conclude that parasite prevalence is far higher in hosts where the parasite has invaded versus hosts in the parasite's native range. The authors further tested this relationship with experimental infections, finding that naive hosts were more susceptible to infection than were hosts from the parasite's native (coevolved) range. Together, these results suggest that hosts have evolved over time to resist parasitism and that parasite invasion may have disproportionate effects on previously naive host populations. This study highlights the potential for cascading effects on local ecosystems, as species ranges shift and parasites are introduced into host populations that lack an evolutionary relationship with them.

## 3 CONCLUSIONS AND FUTURE DIRECTIONS

The papers in this special issue demonstrate the value of investigating marine invasions from an evolutionary perspective and highlight recent technological advances that are key to resolving the uncertainty that often surrounds species invasions. Several papers in this special issue underscore the growing ability of high‐throughput sequencing to elucidate evolutionary dynamics in species invasions (Sherman et al., 2016; Viard, David, & Darling, 2016). While the question of invasion history and source identification has long been an area of active research, the low resolution of traditional molecular tools has hampered our ability to detect subtle differentiation (Estoup & Guillemaud, 2010). This has been a particular issue for marine invasive species, which are often characterized by dynamics such as high dispersal, recent divergence, and complex evolutionary histories that make it difficult to disentangle divergence and admixture at multiple timescales (Darling & Carlton, 2018; Geller et al., 2010). A strong understanding of the evolutionary backgrounds of species invasions, facilitated by high‐throughput sequencing, permits the testing of a number of hypotheses regarding the spread and success of marine invasions. In some systems, admixture (both ancient and recent) may create populations with a rich evolutionary substrate upon which selection pressures in novel environments can act (Keller & Taylor, 2010; Kolbe et al., 2004; Krehenwinkel, Rödder, & Tautz, 2015). In other cases, successful invaders may be genetically depauperate exemplars of the “genetic paradox of invasion,” prompting an examination of the factors that may promote success in the absence of high diversity (Roman & Darling, 2007). As sequencing technologies and analytical approaches improve, we expect to see great improvements in our understanding of the evolutionary pasts—and futures—of marine invasions.

A critical component of success in a novel environment is the ability to reproduce successfully, and papers in this special issue also address the importance of selection on reproductive biology. While selection on reproductive parameters is a major fixture of fundamental evolutionary research, thus far it has not been examined as extensively in marine invasions (Barrett et al. 2008; Manier et al., 2013). As this field of research expands, particularly using careful, well‐controlled physiological experimental designs, we anticipate a wealth of insights into this key stage in the establishment of introduced marine species. Prior work examining the “genetic paradox” in marine invasions has noted the key role that asexual reproduction may play in facilitating the success of highly bottlenecked populations (Roman & Darling, 2007; Gutekunst et al., 2018; North, Pennanen, Ovaskainen, & Laine, 2011). This is especially pertinent to organisms with complex life cycles, notably many species of macroalgae, where these complex life cycles may strongly influence their successful establishment and spread in novel environments (Krueger‐Hadfield et al., 2016). Adopting an analytical framework that explicitly incorporates a species’ reproductive system will greatly improve our understanding of the evolutionary dynamics at play in marine invasions of macroalgae and other species with multiple reproductive options.

Finally, papers in this special issue highlight the importance of evolutionary dynamics in the introduction of marine parasites. While most studies focus on introductions of easily observed, free‐living marine species, the invasion of parasites to systems may be cryptic until they reach high levels of host impact (Lymbery, Morine, Kanani, Beatty, & Morgan, 2014). Host–parasite coevolution represents a canonically powerful selection dynamic, and selection is likely to play a substantial role in the dynamics of introduced parasites and their hosts (Hamilton, Axelrod, & Tanese, 1990; Miller & Vincent, 2008; Wendling, Wegner, & Wendling, 2015). While all introduced species run the risk of founder effects, this may be particularly acute for parasites, which must pass through complex, multi‐host life cycles to establish in a new region. Successful parasites may have to adapt to new hosts to complete their life cycles in a novel region, with potential evolutionary pressures on both parasite and host (Goedknegt et al., 2016). The impact of parasite introductions may be mediated by the evolutionary history of both parasite and host, with potentially extreme effects on native hosts, which may have no evolved defenses against a novel parasite (Peeler, Oidtmann, Midtlyng, Miossec, & Gozlan, 2011). Our current understanding of the impacts of marine parasites is hampered considerably by a dearth of knowledge of native parasite populations. We suggest that a more complete characterization of marine parasite communities will aid in identifying and studying future parasite introductions.

This special issue presents a range of studies speaking to emerging areas of research on the evolutionary biology of marine invasions. Evolution plays a key role in species survival and persistence in new or changing marine environments. Studies of rapid evolution in marine invasions can thus provide fundamental insights into the speed and nature of rapid adaptive change in the ocean, improving our understanding of how marine species more broadly can adapt to changing seas. Moreover, marine invasions can help us generally understand evolutionary processes in real time since introduced populations can serve as natural experiments in evolution, as non‐native species spread and adapt to novel environments. On an applied level, invasion studies can also inform our understanding of the evolutionary traits that facilitate introduction and establishment, improving our ability to predict and prevent future introductions. We propose that an evolutionary perspective should be integral to marine invasion studies and that such a perspective will benefit both fundamental evolutionary biology and the prediction and management of marine invasions.
